# Chrysin Protects against Focal Cerebral Ischemia/Reperfusion Injury in Mice through Attenuation of Oxidative Stress and Inflammation

**DOI:** 10.3390/ijms151120913

**Published:** 2014-11-13

**Authors:** Yang Yao, Li Chen, Jinting Xiao, Chunyang Wang, Wei Jiang, Rongxin Zhang, Junwei Hao

**Affiliations:** 1 Department of Neurology and Tianjin Neurological Institute, Tianjin Medical University General Hospital, Tianjin 300052, China; E-Mails: yaoy1986@gmail.com (Y.Y.); chenli06015105009@126.com (L.C.); xiaojinting1103@163.com (J.X.); wangchunyang0407@gmail.com (C.W.); jiangwei.med@gmail.com (W.J.); 2 Laboratory of Immunology and Inflammation, Research Center of Basic Medical Science, Tianjin Medical University, Tianjin 300070, China; E-Mail: rongxinz@yahoo.com

**Keywords:** chrysin, cerebral ischemia-reperfusion injury, neuroinflammation, oxidative stress

## Abstract

Inflammation and oxidative stress play an important part in the pathogenesis of focal cerebral ischemia/reperfusion (I/R) injury, resulting in neuronal death. The signaling pathways involved and the underlying mechanisms of these events are not fully understood. Chrysin, which is a naturally occurring flavonoid, exhibits various biological activities. In this study, we investigated the neuroprotective properties of chrysin in a mouse model of middle cerebral artery occlusion (MCAO). To this end, male C57/BL6 mice were pretreated with chrysin once a day for seven days and were then subjected to 1 h of middle cerebral artery occlusion followed by reperfusion for 24 h. Our data show that chrysin successfully decreased neurological deficit scores and infarct volumes, compared with the vehicle group. The increases in glial cell numbers and proinflammatory cytokine secretion usually caused by ischemia/reperfusion were significantly ameliorated by chrysin pretreatment. Moreover, chrysin also inhibited the MCAO-induced up-regulation of nuclear factor-kappa B (NF-κB), cyclooxygenase-2 (COX-2), and inducible nitric oxide synthase (iNOS), compared with the vehicle. These results suggest that chrysin could be a potential prophylactic agent for cerebral ischemia/reperfusion (I/R) injury mediated by its anti-inflammatory and anti-oxidative effects.

## 1. Introduction

Stroke is believed to be the second most common cause of death and the main factor leading to long-term disability as a result in irreversible brain injury and loss of neuronal function [1]. Of the three types of stroke (intracerebral hemorrhage, ischemic stroke, and subarachnoid hemorrhage), 70%–80% of cases are ischemic, which is characterized by the occlusion of blood vessels by the formation of an obstructive thrombus or embolus. Ischemia/reperfusion (I/R) injury, which is the cascade of events leading to neuronal injury and death, involves the release of nitric oxide, excitatory amino acids, cytokines, and free radicals, mitochondrial respiratory enzymes damage, the induction of programmed cell death, and microglia activation [2]. Adjunct therapies that increase ischemic tolerance or limit reperfusion injury may extend the therapeutic window or improve the efficacy of reperfusion therapy. Several neuroprotective agents have been investigated in preclinical studies, although none have demonstrated efficacy in clinical practice.

In recent years, the benefits of traditional Chinese medicines exhibiting neuroprotective effects have been increasingly investigated in I/R injury [3]. Chrysin (5,7-dihydroxyflavone), which is a natural flavonoid that is present in honey, bee propolis, and many plant extracts [4,5], is well-known for its various biological activities. Multiple studies have indicated antioxidant [6], antihypertensive [7], antidiabetogenic [8], and anxiolytic functions [9]. In particular, chrysin has the ability to block the cell cycle, induce apoptosis [10], disrupt mitotic spindle formation [11] and inhibit angiogenesis [12], making it a potential candidate as an anticancer drug. Recent, studies have shown that chrysin inhibits the *NF-κB* signaling pathway, providing an underlying mechanism for its anti-inflammatory activity [13,14,15].

However, the therapeutic potential of chrysin in I/R injury is unknown. Thus, the goal of the current study was to assess the neuroprotective capacity of chrysin and the underlying mechanisms in a mouse model of middle cerebral artery occlusion (MCAO) and reperfusion.

## 2. Results and Discussion

### 2.1. Effect of Chrysin on Neurological Deficits, Infarct Volume and Pathomorphological Changes

Chrysin has been implicated as an adjunct therapeutic agent for I/R injury. Multiple previous studies conducted in various animal models, have shown that chrysin exerts anti-inflammatory and neuroprotective effects when administered at doses ranging from 25 to 100 mg/kg/day [6,16,17]. Therefore, in this study, we first evaluated the clinical scores in a mouse model of MCAO following pretreatment with chrysin at the 25, 50, 75, and 100 mg/kg. Clinical scores were lower in mice treated with the 75 or 100 mg/kg doses compared to the control mice, while the 25 and 50 mg/kg doses had no significant effect (data not shown). On the basis of these findings, mice were therefore pretreated with chrysin at doses of 75 mg/kg/day for this study.

**Figure 1:**
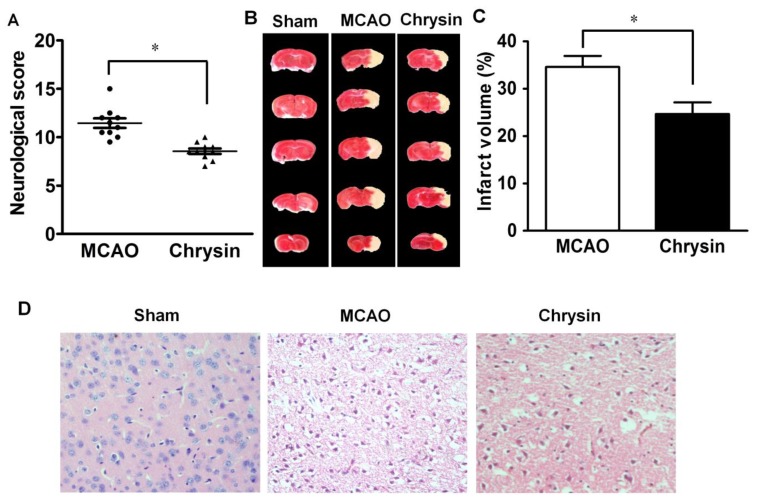
Effect of chrysin on neurological deficits, brain infarct volume and pathomorphological changes in mice with cerebral I/R injury. (**A**) Neurological score was measured in mice that underwent 1 h of ischemia followed by 24 h of reperfusion. Chrysin decreased the neurological score compared to the middle cerebral artery occlusion (MCAO) group; (**B**) Representative coronal brain sections stained with 1% TTC. Mice were subjected to 1 h of ischemia followed by 24 h of reperfusion; the infarct area is white; (**C**) Quantitative analysis of the percentage of brain infarct volume. Chrysin pretreatment diminished the percentage of brain infarct volume and there is a statistical difference compared with the MCAO group; (**D**) H&E staining showed that the normal morphologic features of neurons were present in the sham group. The MCAO group showed the loss of neurons and the presence of multiple vacuolated interspaces. Chrysin significantly ameliorated the damage of neurons that is associated with ischemia in the MCAO group (Magnified 20×). Data are expressed as means ± SEM, * *p* < 0.05, *vs.* MCAO group, *n* = 6.

An examination of neurological function was carried out in mice subjected to 1 h of ischemia followed by 24 h of reperfusion. Compared with the MCAO group, the neurological deficit scores were significantly reduced in mice treated with chrysin (*p* < 0.05). The range of neurological deficit scores for the different groups is shown in Figure 1A. Representative brain slices showed that normal, viable brain tissues were stained deep red, while staining of the infarcted area was pale. No areas of infarction were observed in the sham group. The MCAO group had an obvious rise of infarct volume in comparison with the sham group. TTC staining (2,3,5-tripenyltetrazolium chloride) of brain sections following MCAO and reperfusion showed noticeable damage in the areas supplied by the middle cerebral artery. In comparison with the MCAO group, chrysin pretreatment significantly reduced the infarct volume (Figure 1B,C).

H&E staining of brain tissues subjected to 1 h ischemia followed by 24 h reperfusion revealed neuronal loss and the presence of multiple vacuolated interspaces. Examination of the MCAO group brain sections showed that intact neurons were absent in those areas (Figure 1D). In contrast, the corresponding areas of brain sections from the chrysin pretreatment group showed partial neuronal loss and the presence of intact neurons in between the vacuolated spaces (Figure 1D), thus, indicating that chrysin pretreatment was neuroprotective.

### 2.2. Effect of Chrysin on SOD Activity and MDA Content

**Figure 2:**
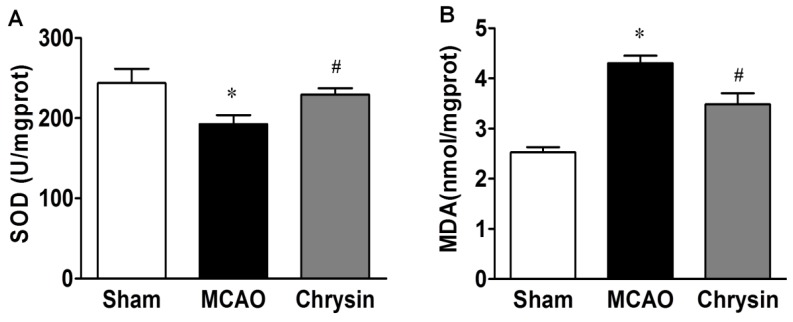
Effect of chrysin on the activity of SOD and the contents of MDA. (**A**) SOD activity were significantly increased in the MCAO group as compared to the sham group, Compared with the MCAO group, the activities of SOD were significantly increased in the chrysin pretreatment group; (**B**) The contents of MDA were significantly increased in the MCAO group as compared to the sham group, while it was significantly decreased in the chrysin pretreatment group. Data are expressed as means ± SEM, * *p* < 0.05, MCAO *vs.* sham group, ^#^ *p* < 0.05, chrysin pretreatment group *vs.* MCAO group, *n* = 6.

Both superoxide dismutase (SOD) activity and malondialdehyde (MDA) levels were measured evaluate the level of oxidative stress associated with I/R injury. Compared with the sham group, SOD activity in the MCAO group was dramatically decreased (*p* < 0.05) and the MDA content increased significantly after I/R injury (*p* < 0.05). Compared with MCAO group, the SOD activity after I/R injury markedly increased, while the MDA content decreased significantly in the chrysin pretreatment group (both *p* < 0.05) (Figure 2). These data suggest that chrysin reduced MCAO-induced oxidative stress.

### 2.3. Effects of Chrysin on Levels of iNOS, COX-2 and NF-κB

**Figure 3:**
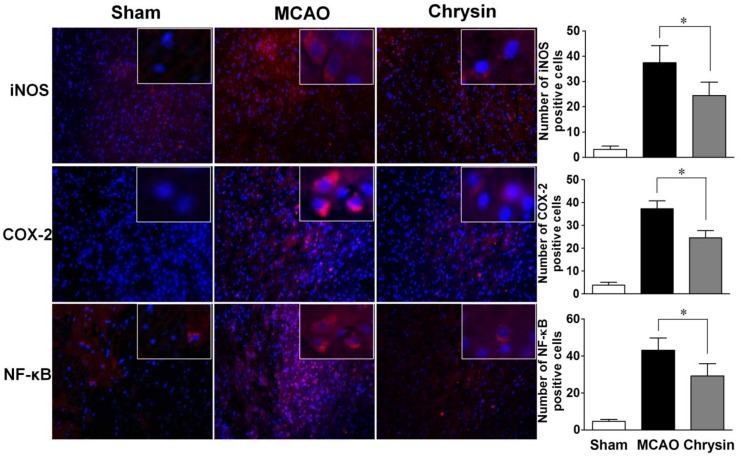
Chrysin pretreatment suppresses the production of *iNOS*, *COX-2* and *NF-κB* in the I/R injured mouse brain. Representative coronal brain sections of the sham group, the MCAO group and the chrysin pretreatment group were stained using immunofluorescence for *iNOS*, *COX-2* and *NF-κB*. The sham group showed negligible staining. However, the expression of *iNOS*, *COX-2* and *NF-κB* was prominent in the MCAO group compared with the sham group, and the chrysin pretreatment group showed moderate staining for *iNOS*, *COX-2* and *NF-κB* (Magnified 20×). Data are expressed as means ± SEM, * *p* < 0.05, *vs.* MCAO group, *n* = 6.

**Figure 4:**
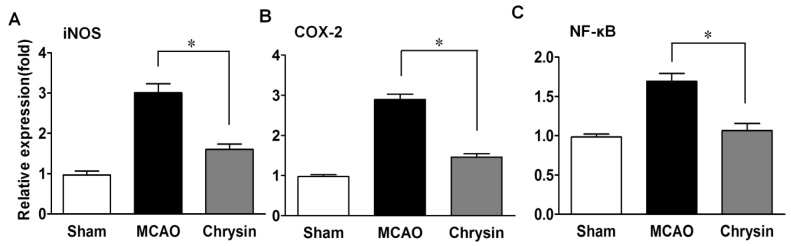
Chrysin pretreatment suppresses the mRNA expression of *iNOS* (**A**), *COX-2* (**B**) and *NF-κB* (**C**) in the MCAO model. Representative mRNA expression of *iNOS*, *COX-2* and *NF-κB* detected by RT-PCR. Compared to the MCAO group, chrysin pretreatment also inhibited the mRNA expression of *iNOS*, *COX-2* and *NF-κB*. Data are expressed as means ± SEM, * *p* < 0.05, *vs.* MCAO group, *n* = 6.

*NF-κB* is known to regulate the expression of *iNOS* and *COX-2*. Immunofluorescence staining showed that the expression levels of *COX-2*, *iNOS* and *NF-κB* were significantly raised in the peri-infarct areas of the brain in the MCAO group (Figure 3). However, pretreatment with chrysin suppressed the expression of *COX-2*, *iNOS* and *NF-κB* compared to mice that did not receive this treatment. Further analyses of mRNA expression in the ischemic-reperfusion brains were carried out with real-time PCR. As shown in Figure 4, the chrysin pretreatment group had markedly reduced mRNA levels of *iNOS, COX-2 and NF-κB* compared with the MCAO group (*p* < 0.05). These data suggest that the anti-inflammatory activity of chrysin is mediated by inhibiting *NF-κB* activation and *iNOS* and *COX-2* gene expression.

### 2.4. Effect of Chrysin on the Expression of GFAP and Iba-1

**Figure 5:**
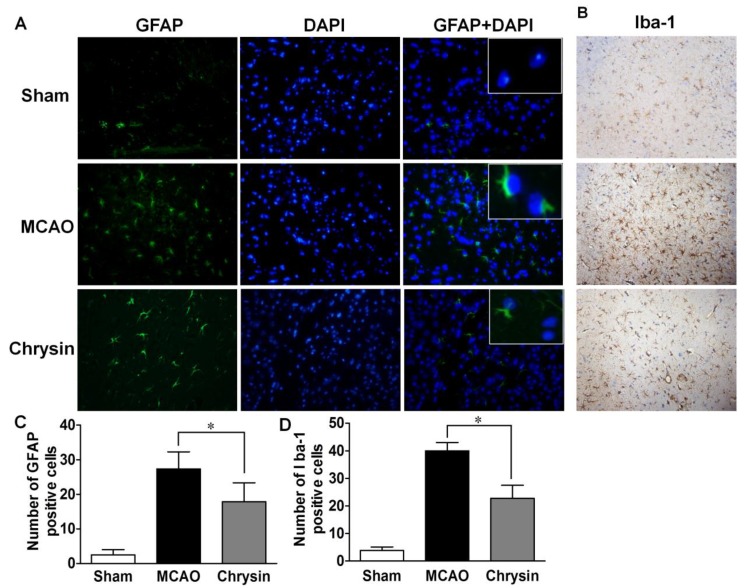
Chrysin suppressed the expression of GFAP and Iba-1. (**A**) Immunofluorescence staining in cortical brain sections for the sham group, the MCAO group and the chrysin pretreated group show the expression of GFAP; (**A**,**C**) The sham group showed negligible GFAP-positive cells. However, the expression level of GFAP was prominent for the MCAO group compared with the sham group, while the chrysin pretreatment group showed moderate expression of GFAP levels. GFAP-positive cells and DAPI-positive nuclei were co-localized (Magnification 40×); (**B**,**D**) The expression of Iba-1 was observed in the all three treatment groups. The MCAO group showed increased gliosis compared to the sham group, while the chrysin pretreatment group showed a moderate expression of Iba-1 (Magnification 20×). Data are expressed as means ± SEM, * *p* < 0.05, *vs.* MCAO group, *n* = 6.

Neuronal death in cerebral I/R injury is associated with astrocytosis and microgliosis. The numbers of GFAP- and Iba-1-positive cells were significantly increased in the ischemic-reperfusion brain sections in the MCAO group (Figure 5), compared to the sham-operated group. Compared with the MCAO group, there was a marked decrease in GFAP and Iba-1 expression in the chrysin pretreated group, suggesting that chrysin inhibits astrocytosis and microgliosis.

### 2.5. Effects of Chrysin Treatment on Cytokine Profiles

**Figure 6:**
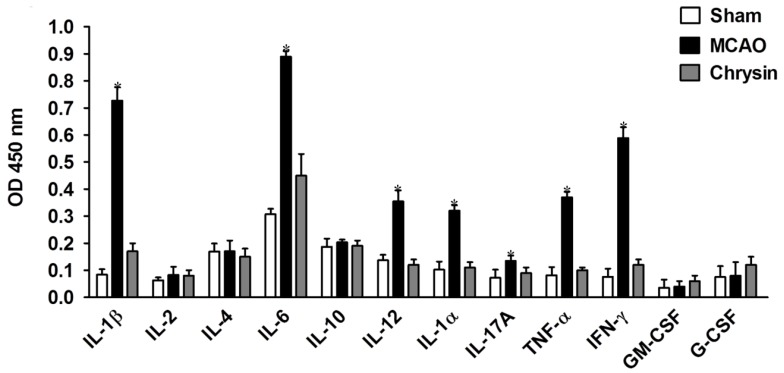
Chrysin reduced the secretion of proinflammatory cytokines. The brain tissue supernatants in mice that underwent 1 h of ischemia followed by 24 h of reperfusion were measured by ELISA. IL-1β, IL-6, IL-12, IL-1α, IL-17A, IFN-γ and TNF-α were inhibited, however, IL-2, IL-4, IL-10, G-CSF and GM-CSF showed no significant changes. The results are presented as mean OD ± SEM. * *p* < 0.05, *vs.* MCAO group, *n* = 6.

Semi-quantitative analysis of cytokine levels in the brain tissues of mice subjected to 1 h ischemia followed by 24 h reperfusion was performed using multi-cytokine ELISA kits. As shown in Figure 6, the production of proinflammatory cytokines (IL-1β, IL-6, IL-12, IL-1α, IL-17A, IFN-γ and TNF-α) in the chrysin pretreatment group was significantly reduced compared to that in the MCAO group (*p* < 0.05). However, there were no marked differences in the levels of IL-2, IL-4, IL-10, G-CSF, and GM-CSF among the three groups (Figure 6). These data further confirm the anti-inflammatory properties of chrysin.

### 2.6. Discussion

In the present study, we used a temporary MCAO and reperfusion mouse model to determine the effects of chrysin following acute ischemic stroke. The intraluminal suture method is the most commonly adopted animal model of ischemic stroke used to study the neuroprotective effect of drugs. Our results show that infarct size, inflammation, and oxidative stress were reduced in this model by chrysin pretreatment.

Histology can be used to link the development of the I/R lesion and the extent of neural protection conferred by pharmacological intervention. Infarction volume and neurological deficits are key factors for evaluating the consequences of stroke [18] with TTC and H&E staining employed to identify the infarcted areas. In the current study, the infarct regions in the MCAO group were clearly visible; however, the size of the affected regions and neuronal necrosis was significantly reduced by chrysin pretreatment.

Accumulating recent evidence has shown that the overproduction of oxygen free radicals, such as superoxide anions, hydroxyl radicals and hydrogen peroxide, during reperfusion plays an important role in acute ischemic stroke. Furthermore, considerable evidence has linked the production of reactive oxygen species (ROS) and subsequent oxidative damage to the pathogenesis of I/R injury [19]. The mechanism of this effect may involve the stimulation of a radical ripple effect or activation of a signaling pathway that promotes damage to cellular macromolecules, leading to irreversible neuronal injury and cell death [20]. Based on the findings of the present study, we conclude that the antioxidative properties of chrysin are due in part to the inhibition of *iNOS* and *COX-2* expression. In addition, the anti-inflammatory properties of chrysin were further confirmed by its effective inhibition of pro-inflammatory *NF-κB* activity. Thus, the down-regulation of *iNOS* and up-regulation of *COX-2* provides a molecular basis for the neuroprotective effects of chrysin in I/R injury. Furthermore, SOD is believed to be the critical scavenger enzyme hindering tissue injury caused by peroxidase reactions. In addition, expression levels of MDA, a toxic final product of lipid peroxidation; is inversely linked to the ability of SOD to reduce the rate and extent of lipid peroxidation free radical productions [21]. Our results show the MDA content was markedly lower and the SOD activity slightly increased in the chrysin pretreated MCAO group compared with the vehicle-treated MCAO group. Overall, these results indicate that the potential of chrysin for the treatment of I/R injury due to its anti-oxidative properties.

Reactive gliosis, which is associated with ischemic stroke involves both astrocytes and microglia, is an important component of the cellular and molecular pathways involved in stroke-induced destructive responses. In our study, I/R injury increased GFAP and Iba-1 expression, while the expression of these markers was reduced in mice pretreated with chrysin. These observations suggest that chrysin inhibits the proliferation of glial cells, further explaining its neuroprotective properties.

Acute neuronal cell death after the onset of I/R injury is also associated with inflammatory mechanisms. Inflammatory cascades are initiated by energy depletion and necrotic neuronal cell death in the local ischemic area; therefore, inhibitors of proinflammatory cytokines are likely to have protective benefits during I/R injury. In this study, we observed the inhibition of IL-1β, IL-6, IL-12, IL-1α, IL-17A, IFN-γ, and TNF-α following chrysin pretreatment. In contrast, no significant changes in IL-2, IL-4, IL-10, G-CSF, and GM-CSF were observed in response to chrysin pretreatment. These data demonstrate that the anti-inflammatory effects of chrysin are associated with the regulation of inflammatory cytokine secretion.

## 3. Experimental Section

### 3.1. Animals and Reagents

Male C57BL/6 mice (aged 10–12 weeks) were purchased from the Academy of Military Medical Science (Beijing, China). The mice were housed under temperature control and a 12-h light-12-h dark cycle with food and water provided *ad libitum*. The experiments were performed according to national regulations and approved by the Animal Experiments Ethical Committee of Tianjin Medical University General Hospital. Mice were acclimated to these conditions for one week following their arrival and prior to the start of the experiments. Chrysin (>98% purity, by high performance liquid chromatography analysis) was purchased from Nanjing TCM Institute of Chinese Materia Medica (Nanjing, China). It was freshly prepared in phosphate-buffered saline (PBS) containing 2% (*v*/*v*) dimethylsulfoxide (DMSO). Chrysin was administered by oral gavage at a dose of 75 mg/kg body weight per day for 7 days. Control animals received the same volume of the vehicle (PBS/2% DMSO) using the same regimen.

### 3.2. MCAO Model and Clinical Evaluation

Animals were randomly separated into three groups of (6–10 mice per group): Group I, sham-operated group (vehicle-treated); Group II, MCAO group (vehicle-treated); Group III, chrysin pretreatment MCAO group. Focal cerebral ischemia was simulated by occlusion of the left middle cerebral artery based on the methods described by Longa *et al.* [22]. Briefly, the mice were anesthetized with chloral hydrate (30 mg/kg, intraperitoneally). A midline neck incision was then made to expose, the left common carotid artery, the external carotid artery and the internal carotid artery, which were isolated and ligated. A monofilament coated with silicone rubber (Shadong, Beijing, China) was inserted into the internal carotid artery (9–10 mm) through the common carotid artery until a mild resistance was felt, indicating occlusion of all blood flow from the posterior cerebral artery, the internal carotid artery, and the anterior artery. One hour after the induction of ischemia, the monofilament was removed to restore blood flow. The sham-operated group mice underwent all surgical procedures with the exception of monofilament insertion into the internal carotid artery. The body temperature of the mice was maintained at 37.0 ± 0.5 °C during surgery and mice were kept in a well-ventilated room at 25 ± 3 °C in individual cages, with the provision of food and water, until they regained full consciousness. Neurological function was evaluated in each mouse group 24 h after the MCAO and reperfusion using the modified Neurological Severity Score (mNSS) [23].

### 3.3. Infarct Volume Analysis

After clinical scoring, the brains of the MCAO and reperfusion mice were immediately sliced into coronal sections (2 mm thick) from the frontal tips using scalpels. The sections were stained with 1% 2,3,5-tripenyltetrazolium chloride (TTC; Sigma, St. Louis, MO, USA) and immersed in normal saline at 37 °C for 20 min. Brain sections were then fixed in 4% paraformaldehyde at 4 °C overnight before being photographed. Viable tissues stain deep red based on intact mitochondrial function, while infarcts remain unstained. The infarcted regions in each section were evaluated using Image-Pro^®^ Plus v 4.0 image analysis software (Media Cybernetics, Washington, DC, USA). The total infarct volume was calculated as the sum of the infarct volume of each section. To compensate for the effect of brain edema, the infarct volume percentage was calculated as follows: infarct area × {1 − [(ipsilateral hemisphere area − contralateral hemisphere area)/contralateral hemisphere]} [24].

### 3.4. Pathological/Histological Analysis

After 1 h of ischemia followed by 24 h of reperfusion, the mice were anesthetized with chloral hydrate (30 mg/kg, intraperitoneal) and transaortically perfused with 4% paraformaldehyde dissolved in 0.1 M phosphate-buffered saline (PBS, pH 7.4, 4 °C). The brains were removed and postfixed in 4% paraformaldehyde overnight at 4 °C. After being cut into successive, paraffin-embedded coronal sections (6 μm), the brain sections were stained with hematoxylin and eosin (H&E). The pathological and histological changes were observed through a light microscope (Olympus, Tokyo, Japan) magnification at 20× and photographed.

### 3.5. Determination of SOD Activity and MDA Level

After I/R injury as described, brains were collected to determine SOD activity and MDA levels as indicators of oxidative stress. The brains were washed, weighed and then homogenized in ice-cold saline (9 volumes) for 20 min to prepare a 10% (*w*/*v*) homogenate. The homogenate was then centrifuged at 4000 rpm/min for 10 min at 4 °C. SOD activity and MDA levels were then measured by assay kits (A001 and A003; Nanjing Jiancheng Bioengineering Institute, Nanjing, China) in accordance with the manufacturers’ instructions. The assay results were normalized to the protein concentration in each sample, and expressed as U/mg protein or nmol/mg protein.

### 3.6. Immunofluorescence Analysis of iNOS, COX-2, NF-κB and GFAP

Paraffin-embedded coronal brain sections (6 μm) were subjected to deparaffinization, rehydration, and underwent a microwave oven antigen retrieval (microwave method). The brain sections were incubated overnight at 4 °C with the following primary antibodies: rabbit anti-mouse *iNOS* antibody (1:100 dilution, Zhongshan Goldenbridge Biotechnology, Beijing, China), rabbit anti-mouse *COX-2* antibody (1:100 dilution, Zhongshan Goldenbridge Biotechnology), mouse anti-mouse *NF-κB* antibody (1:50 dilution, Zhongshan Goldenbridge Biotechnology) or rabbit anti-mouse glial fibrillary acidic protein (GFAP) antibody (1:2000 dilution, Abcam Biotechnology, Cambridge, MA, USA). The slides were rinsed with cold PBS in order to remove the unbound antibodies. Sections were then incubated with IgG secondary antibody (1:2000 dilutions, goat anti-rabbit, Abcam Biotechnology) for 1 h at room temperature followed by 4',6-diamidino-2-phenylindole (DAPI) for 5 min at room temperature. Finally, the sections were mounted with mounting media, cover-slipped and air-dried. Cells stained for *iNOS*, *COX-2*, *NF-κB* and GFAP in the core ischemic wound of the cerebral tissues were randomly analyzed in 10 sections of each brain under high magnification (20× or 40×). The results were presented as mean ± standard error of the mean (SEM).

### 3.7. Immunohistochemical Detection of Iba-1

Brain sections were exposed to 3% hydrogen peroxide for 10 min to destroy endogenous peroxidases activity and then blocked with bovine serum albumin for 30 min. Sections were subsequently incubated (in PBS) overnight at 4 °C with rabbit anti-mouse ionized calcium binding adapter molecule (Iba-1) antibody (1:1000 dilution, Abcam Biotechnology) diluted in a solution of 0.3% Triton X-100. Sections were then incubated with anti-rabbit IgG secondary antibody for 1 h at room temperature. The primary antibody was replaced with PBS in negative control sections. After washing in PBS, immunoreactivity was detected with 3,3'-diaminobenzidine tetrahydrochloride (DAB) and counterstained with hematoxylin. Finally, the brain sections were dehydrated in a graded ethanol series, mounted in xylene and coverslipped. The results were expressed as the number of positive cells per mm^2^ tissue section counted in 10 randomly selected visual fields under high magnification (20×).

### 3.8. Quantitative Real-Time PCR

Total RNA was extracted from the ischemic hemisphere using Trizol reagent (Invitrogen, Carlsbad, CA, USA) according to the manufacturer’s instructions. The concentration of RNA was quantified by ultraviolet spectrophotometry at 260/280 nm. cDNA was transcribed using QuantiTect Reverse Transcription Kit (Qiagen, Hilden, Germany) in accordance with the manufacturer’s instructions. The primer sequences used to measure gene expression were: *iNOS* sense [25,26], GGA ATC TTG GAG CGA GTT GTG GAT, and antisense, CCT CCA ATC TCT GCC TAT CCG TCT; *COX-2* sense [27,28], ATC ATA AGC GAG GAC CTG GGT TCA C, and antisense, TCT CTG GGA TGT GAG GAG GGT AGA T; and *NF-κBp65* sense [29,30], ACC GTC CTC TGC CCC AGT AT and antisense, GGC TTC CGA CAG CGT GCC TT. In addition, *β-actin* was used as an internal control. Quantitative real-time polymerase chain reaction (PT-PCR) analysis of *iNOS*, *COX-2* and *NF-κB* mRNA expression was performed using SYBR green mix (Newbioindustry, Beijing, China) and calculated using the 2^−ΔΔ*T*h^ method.

### 3.9. Measurement of Cytokines

Cytokines in supernatants from ischemic brain tissue cultures were measured with a quantitative enzyme-linked immunosorbent assay (ELISA) using the Multi-Analyte ELISArray Kit (Qiagen, Duesseldorf, Germany). The kit detects 12 cytokines simultaneously (IL-1α, IL-1β, IL-2, IL-4, IL-6, IL-10, IL-12, IL-17A, TNF-α, interferon-γ (IFN-γ), granulocyte colony-stimulating factor (G-CSF) and granulocyte-macrophage colony-stimulating factor (GM-CSF)) using an ELISA protocol conducted under uniform conditions. according to the manufacturer’s instructions. Determinations were performed in duplicate on individual mouse brains (*n* = 6 mice per group) and the results were expressed as mean OD value.

### 3.10. Statistical Analysis

SPSS 17.0 analysis software (SPSS. Inc, Chicago, IL, USA) was used to compare the differences between each group. The processed data were presented as mean ± SEM. For all statistical analyses, the level of significance was set at *p* < 0.05.

## 4. Conclusions

In summary, the findings of this study suggest that chrysin is a potentially useful prophylactic agent for I/R injury due to its anti-inflammatory and anti-oxidative properties, and may provide a potential promising new therapeutic strategy for acute ischemic stroke.
